# Prediction of Immune-Related Adverse Events Induced by Immune Checkpoint Inhibitors With a Panel of Autoantibodies: Protocol of a Multicenter, Prospective, Observational Cohort Study

**DOI:** 10.3389/fphar.2022.894550

**Published:** 2022-06-01

**Authors:** Iñigo Les, Inés Pérez-Francisco, María Cabero, Cristina Sánchez, María Hidalgo, Lucía Teijeira, Virginia Arrazubi, Severina Domínguez, Pilar Anaut, Saioa Eguiluz, Iñaki Elejalde, Alberto Herrera, Mireia Martínez

**Affiliations:** ^1^ Internal Medicine Department, Navarra University Hospital, Pamplona, Spain; ^2^ Autoimmune Diseases Unit, Internal Medicine Department, Navarra University Hospital, Pamplona, Spain; ^3^ Bioaraba Health Research Institute, Breast Cancer Research Group, Vitoria-Gasteiz, Spain; ^4^ Bioaraba Health Research Institute, Clinical Trials Platform, Vitoria-Gasteiz, Spain; ^5^ Osakidetza Basque Health Service, Araba University Hospital, Department of Internal Medicine, Vitoria-Gasteiz, Spain; ^6^ Osakidetza Basque Health Service, Araba University Hospital, Department of Medical Oncology, Vitoria-Gasteiz, Spain; ^7^ Medical Oncology Department, Navarra University Hospital, Pamplona, Spain; ^8^ Osakidetza Basque Health Service, Araba University Hospital, Department of Immunology, Vitoria-Gasteiz, Spain; ^9^ Bioaraba Health Research Institute, Lung Cancer Research Group, Vitoria-Gasteiz, Spain

**Keywords:** immune checkpoint inhibitors, immune-related adverse events, cancer, autoantibodies, prospective cohort study, multicenter

## Abstract

**Introduction:** Immune checkpoint inhibitor (ICI) therapy is markedly improving the prognosis of patients with several types of cancer. On the other hand, the growth in the use of these drugs in oncology is associated with an increase in multiple immune-related adverse events (irAEs), whose optimal prevention and management remain unclear. In this context, there is a need for reliable and validated biomarkers to predict the occurrence of irAEs in patients treated with ICIs. Thus, the main objective of this study is to evaluate the diagnostic performance of a sensitive routinely available panel of autoantibodies consisting of antinuclear antibodies, rheumatoid factor, and antineutrophil cytoplasmic antibodies to identify patients at risk of developing irAEs.

**Methods and Analysis:** A multicenter, prospective, observational, cohort study has been designed to be conducted in patients diagnosed with cancer amenable to ICI therapy. Considering the percentage of ICI-induced irAEs to be 25% and a loss to follow-up of 5%, it has been estimated that a sample size of 294 patients is required to detect an expected sensitivity of the autoantibody panel under study of 0.90 with a confidence interval (95%) of no less than 0.75. For 48 weeks, patients will be monitored through the oncology outpatient clinics of five hospitals in Spain. Immune-related adverse events will be defined and categorized according to CTCAE v. 5.0. All the patients will undergo ordinary blood tests at specific moments predefined per protocol and extraordinary blood tests at the time of any irAE being detected. Ordinary and extraordinary samples will be frozen and stored in the biobank until analysis in the same autoimmunity laboratory when the whole cohort reaches week 48. A predictive model of irAEs will be constructed with potential risk factors of immune-related toxicity including the autoantibody panel under study.

**Ethics and Dissemination:** This protocol was reviewed and approved by the Ethical Committee of the Basque Country and the Spanish Agency of Medicines and Medical Devices. Informed consent will be obtained from all participants before their enrollment. The authors declare that the results will be submitted to an international peer-reviewed journal for their prompt dissemination.

## Introduction

### Cancer and Immune Checkpoint Inhibitor Therapy

In recent years, immune checkpoint inhibitors (ICIs) have been widely used as first- and second-line treatments for several types of cancer: melanoma, renal cell carcinoma, non-small cell lung carcinoma, urothelial carcinoma, head and neck squamous cell carcinoma, and Hodgkin’s lymphoma (Postow et al., 2015; Wilson et al., 2018). The mechanism of action of these drugs consists of blocking certain immune checkpoints within the immune system, which physiologically inhibit the activation of T cells seeking to trigger an enhanced anti-tumor immune response.

T-cell activation depends on the existence of two effector signals: 1) an antigen-specific signal, which consists of the recognition through the T-cell receptor (TCR) of antigens presented by an antigen-presenting cell involving the class II major histocompatibility complex (Hennecke and Wiley, 2001); and 2) a second non-antigen-specific signal known as antigenic co-stimulation, which consists of the interaction between the CD28 receptor (located on the surface of virgin T cells) and the B7 ligand expressed by antigen-presenting cells. Once activated, T cells express cytotoxic T lymphocyte-associated antigen 4 (CTLA-4), an inhibitory molecule homologous to CD28 that also binds to B7 but with a higher affinity than CD28, thereby acting as a physiological brake on T-cell activation through the loss of the co-stimulatory signal (Sharpe, 2009). Ipilimumab, approved for the treatment of metastatic melanoma (Robert et al., 2011), and tremelimumab, pending approval for the treatment of advanced non-small cell lung carcinoma (Johnson et al., 2021), are both monoclonal antibody antagonists that target the CTLA-4 protein.

Another well-characterized immune checkpoint is programmed death cell protein 1 (PD-1), which is expressed on T, B and natural killer cells, and binds to PD-L1 and PD-L2 ligands on various tumor cells. PD-1 and its ligands, PD-L1/2, are inhibitory homologues of CD28 and B7, respectively, and hence, their interaction inhibits T-cell proliferation, promotes central and peripheral T-cell tolerance, and decreases tumor cell apoptosis (Zhang et al., 2004). Furthermore, the PD-1/PD-L pathway, which acts synergistically with CTLA-4, is crucial for the attenuation and termination of the immune response, and therefore, its dysfunction is associated with autoimmune phenomena (Sharpe et al., 2007). Currently approved PD-1/PD-L1 checkpoint inhibitors on the market include PD-1 antibodies such as nivolumab and pembrolizumab, and PD-L1 antibodies such as atezolizumab, durvalumab and avelumab.

### Side Effects of Immune Checkpoint Inhibitor Therapy: Immune-Related Adverse Events and Possible Predisposing Factors

By unblocking certain immune regulatory mechanisms, ICIs can exacerbate pre-existing autoimmune diseases (ADs) (Abdel-Wahab et al., 2018), whose prevalence is estimated to be between 3 and 8% in the general population (Davidson and Diamond, 2001; Cooper and Stroehla, 2003; Tobón et al., 2012), or induce *de novo* immune-related adverse events (irAEs) (De Velasco et al., 2017; Postow et al., 2018). Descriptions have been published of a wide variety of irAEs, both organ-specific (hypophysitis, colitis, pneumonitis, encephalitis, thyroiditis, dermatitis-rash, vitiligo, arthritis, hepatitis, myasthenia gravis) and systemic [Sjögren’s syndrome, giant cell arteritis, systemic lupus erythematosus (SLE), remitting seronegative symmetrical synovitis with pitting edema (RS3PE) syndrome, polymyalgia rheumatica, systemic vasculitis, antiphospholipid syndrome], which hamper treatment in a significant percentage of patients with an established indication for ICIs (Spain et al., 2016). On the other hand, patients with a history of ADs, some of which are considered risk factors for cancer (Trallero-Araguás et al., 2012; Bernal-Bello et al., 2017; Lazzaroni et al., 2017; Yang et al., 2017), have been systematically excluded from pivotal clinical trials on ICIs (Haanen et al., 2020). Thus, experience with the use of ICIs in patients with ADs is scarce and comes from post-marketing observational studies (Johnson et al., 2016; Donia et al., 2017; Menzies et al., 2017; Tang et al., 2021). Despite this barrier to patients with ADs accessing treatment with ICIs, several studies suggest that the response rate is higher in patients with irAEs than those without irAEs (Attia et al., 2005; Teulings et al., 2015; Judd et al., 2017; Fujii et al., 2018; Kostine et al., 2018; Maher et al., 2019; Hussaini et al., 2021). In contrast, immunosuppressive treatment aimed at controlling irAEs may reduce the anti-tumor effect of ICIs, by “immune dampening” (Weber et al., 2016; Kim et al., 2017; Bai et al., 2021). Overall, there is a need for better clinical and immunological characterization of patients who develop irAEs.

Besides the risk of immune-related toxicity due to pre-existing ADs, other factors that may predict the occurrence of irAEs in patients receiving ICIs have yet to be identified. From a pathogenic point of view, ADs arise from an excessive response of the adaptive immune system against self-expressed antigens (autoantigens), resulting in the proliferation of B cells and generation of autoantibodies. Specifically, these autoantibodies are synthesized by memory B cells and long-lived plasma cells because of their interaction with follicular helper CD4 T (Tfh) cells in the germinal centers of secondary lymphoid organs, with the involvement of the PD-1 pathway as a promoter (Zamani et al., 2016). Apart from acting as an inhibitory signal in the T response, PD-1 also modulates the selection and survival of B cells in germinal centers, which is essential for the genesis of long-lived plasma cells (Good-Jacobson et al., 2010) through Tfh cells, which are responsible for the maturation and activation of B cells (Eivazi et al., 2016). Indeed, it has been suggested that the proportion of circulating PD-1-expressing Tfh cells is an indicator of aberrant germinal center activity leading to B hyperactivation and autoantibody generation in SLE patients (Choi et al., 2015; Xu et al., 2015). The frequency of follicular regulatory T cells, which in turn inhibit Tfh proliferation, correlates with anti-DNA levels and lupus activity (Xu et al., 2017). Nonetheless, this PD-1-mediated interaction between B cells and T cells (namely, Tfh cells) depends on a complex and subtle balance, and any dysfunction in this balance may cause autoimmune phenomena. Blockade of PD-1 and PD-L1 (but not PD-L2) promotes the expansion of the Tfh population and the antigen-specific humoral response. PD-1 deficiency leads to a lupus-like syndrome involving arthritis and proliferative glomerulonephritis (Nishimura et al., 1999). In murine models of lupus nephritis, treatment with PD-1 inhibitors reduces CD4+ PD1+ cell count and overall mortality rate, while PD-L1 inhibition increases inflammatory cytokines and autoantibody titers and accelerates renal damage (Kasagi et al., 2010). The presence of (endogenous) anti-PD-1 autoantibodies has also been reported to be associated with increased SLE activity, especially in newly diagnosed patients (Shi et al., 2017). Although all these data suggest the involvement of PD-1 in B-cell-orchestrated humoral immunity, it is currently unknown whether the mechanism by which ICIs lead to irAEs depends on the pre-existence or *de novo* generation of autoantibodies as a preliminary step for the development of toxicity (Cooling et al., 2017; Läubli et al., 2017; Osorio et al., 2017; Yoest, 2017). A further difficulty is that potentially pathogenic autoantibodies may be present in patients with irAEs but at lower levels than those commonly used as a cut-off point for clinical purposes (Yoest, 2017).

### Diagnostic Tests Used in Autoimmune Diseases: Antinuclear Antibodies, Rheumatoid Factor, and Antineutrophil Cytoplasm Antibodies

Among the various types of autoantibodies employed in clinical practice, antinuclear antibodies (ANAs) are the most widely used. These are a heterogeneous group of autoantibodies directed against one or more antigens usually located in the cell nucleus (DNA, nucleosomes, histones, ribonuclear proteins, etc.) but also occasionally in the cytoplasm (actin, vimentin, cytokeratins, histidyl-tRNA synthetase). Due to their high sensitivity, ANA detection is considered the first level test for laboratory diagnosis of ADs with systemic involvement (Agmon-Levin et al., 2014). The measurement of ANAs should be guided by clinical suspicion since as much as 5% of the asymptomatic adult population tests positive for ANAs at a titer of 1:160 (Gordon et al., 2018). Nowadays, the gold standard method for ANA screening is the indirect immunofluorescence assay (IFA), which has the advantage of providing immunofluorescence patterns suggestive of specific antibodies [see International Consensus on ANA Patterns (ICAP) in website www.ANApatterns.org (Damoiseaux et al., 2015; Chan et al., 2022)]. In the event of ANA positivity and depending on the IFA pattern, it is recommended to test for double-stranded DNA (dsDNA) and extractable nuclear antigens (ENAs), which are associated with certain ADs or some of the unique clinical manifestations of such diseases. Both ANAs and some types of ENAs (e.g., anti-Ro antibodies, specific but not exclusive to Sjögren’s syndrome, or anti-M2 antibodies, specific to primary biliary cirrhosis) may precede the diagnosis of a particular AD by several years (Jones, 2000; Jonsson et al., 2013; Hu and Deng, 2014; Theander et al., 2015). In the case of SLE, a progressive accumulation of increasingly specific autoantibodies has been described, preceding disease onset by years in individuals who are still asymptomatic (Arbuckle et al., 2003; Olsen and Karp, 2014). A working hypothesis is that ICIs, by removing the brake on inhibitory proteins, could at the same time accelerate the process of autoantibody aggregation observed in previous studies.

In recent years, one of the best characterized irAEs has been inflammatory arthritis (Belkhir et al., 2017; Kostine et al., 2018; Smith and Bass, 2019). Some expert groups have proposed specific algorithms for patients treated with ICIs who develop inflammatory arthritis recommending the measurement of rheumatoid factor (RF) and anti-citrullinated peptide antibodies (ACPAs) (Naidoo et al., 2017), both biomarkers with proven diagnostic and prognostic value in rheumatoid arthritis. Rheumatoid factors, generally of the immunoglobulin M isotype, antibodies against the constant fraction of immunoglobulin G, are more sensitive than ACPAs for the diagnosis of rheumatoid arthritis (75–80% versus 50–75%) and correlate with severe and extra-articular forms of the disease. In contrast, ACPAs offer higher specificity, in some series exceeding 90% (Nishimura et al., 2007; Payet et al., 2014), which is especially useful in the differential diagnosis of early or undifferentiated polyarthritis (Avouac et al., 2006; Whiting et al., 2010). In patients with rheumatoid arthritis, increased soluble PD-1 is known to be associated with disease activity, autoantibody titers (both RF and ACPA) and progression of radiological damage (Greisen et al., 2014). Nonetheless, in other studies including patients with ICI-induced inflammatory arthritis, ANA, RF or ACPA positivity is inconsistent. Therefore, the hypothesis that immune-related arthritis is mediated by autoantibodies remains to be confirmed.

Another group of autoantibodies useful in the diagnosis of systemic ADs are antineutrophil cytoplasmic antibodies (ANCAs) for systemic necrotizing small vessel vasculitis, also called ANCA-associated vasculitis. As with ANA and ENA screening, two complementary laboratory techniques are widely used for the detection of ANCAs: 1) IFAs (Savige et al., 2005), which are more sensitive and, in case of positivity, provide patterns with clinical correlates that add diagnostic value to the result, while allowing the exclusion of atypical ANCA patterns suggestive of conditions other than vasculitis (ANCA induction by drugs or infections, ulcerative colitis, autoimmune hepatitis, primary sclerosing cholangitis); and 2) enzyme-linked immunosorbent assays (ELISAs), which are a more specific method to confirm or rule out reactivity to disease-relevant target antigens associated with unique clinical forms of ANCA vasculitis and to monitor treatment response and risk of recurrence. There is evidence that certain CTLA-4 and PD-1 polymorphisms may promote T-cell hyperreactivity and contribute to the pathogenesis of some vasculitides (Slot et al., 2008). It has also been reported that dysfunction of the PD-1/PDL-1 pathway may be involved in the development of forms of vasculitis other than those associated with ANCAs (Watanabe et al., 2017) or inflammatory vasculopathy in general (Weyand et al., 2018). In addition, an atypical ANCA pattern, present in certain ADs of the digestive tract, could be useful as a marker for some of the most common irAEs such as colitis, hepatitis, and cholangitis.

### Summary

We are currently witnessing an exponential increase in publications on irAEs; most of them focus, however, on identifying predictors of efficacy rather than toxicity (Hopkins et al., 2017). Nevertheless, recent years have seen the emergence of several methods seeking to achieve early detection of irAEs (Patil et al., 2018; Nakamura, 2019; von Itzstein et al., 2020), namely, analysis of lymphocyte subpopulations (Das et al., 2018), autoantigen microarrays (Gowen et al., 2018; Tahir et al., 2019), molecular omics data (Jing et al., 2020), gene signatures and polymorphisms (Friedlander et al., 2018; Abdel-Wahab et al., 2021), and the gut microbiome (Dubin et al., 2016). All these still experimental tools share the drawback of a high level of complexity, limited availability, and high cost. In parallel, other more accessible risk factors and markers have been suggested, namely, female sex (Valpione et al., 2018), total white blood cell and lymphocyte counts (Fujisawa et al., 2017), peripheral eosinophilia (Chu et al., 2020), sarcopenia (Daly et al., 2017), vitamin D deficiency (Bersanelli et al., 2017) and levels of more easily measurable interleukins (ILs) such as IL-6, IL-17 and IL-10 (Sun et al., 2008; Tarhini et al., 2015). Overall, there is a need for better clinical and immunological characterization of patients who develop irAEs, especially those with pre-existing ADs. Despite systematization efforts (Haanen et al., 2018; Schneider et al., 2021), it remains unclear how best to manage irAEs, and to tackle this question, there is a need to thoroughly understand the duality between the anti-tumor and the immunogenic effects of ICIs.

For all these reasons, the AUTENTIC study aims to investigate the usefulness of a sensitive, accessible, and inexpensive panel of autoantibodies, consisting of ANAs, RF, and ANCAs, to detect patients at risk of developing irAEs induced by ICIs. We also aim to describe the autoantibody profile, the potential early appearance or aggregation of autoantibodies over time, and the impact of irAEs on cancer-related survival of patients receiving ICIs.

## Methods and Analysis

### Objectives

#### Primary Objectives

• To identify predictive factors of irAEs at week 48 after the initiation of ICI therapy
• To evaluate the diagnostic properties (sensitivity, specificity, positive predictive value, negative predictive value, likelihood ratios) of a predictive model of irAEs.

#### Secondary Objectives

• To describe the profile of detectable autoantibodies over a 48-weeks follow-up in patients with cancer treated with ICIs, regardless of whether they develop irAEs
• To compare irAE-free survival among patients positive for autoantibodies before ICI initiation, patients positive for autoantibodies after ICI initiation and patients negative for autoantibodies throughout follow-up
• To analyze cancer-related survival in patients treated with ICIs who develop irAEs and compare it with that in patients who do not develop irAEs
• To investigate the potential early appearance and/or aggregation of autoantibodies over time up to the time of irAEs diagnosis.

This study protocol corresponds to the first phase of the AUTENTIC project, which will be followed by an external validation of a predictive model of irAEs with prospective data in an independent cohort of patients.

### Endpoints

The primary endpoint of the study will be the proportion of patients developing at least an irAE at week 48 after the initiation of ICI therapy. An irAE was defined as any symptom, sign, syndrome, or disease caused by an immune-activating mechanism during the administration of an ICI once other causes such as an infectious disease or tumor progression have been ruled out.

Key secondary endpoints will be irAE-free survival, progression-free survival, cancer-related survival and overall survival. Other secondary outcomes will be proportion of ICI withdrawal due to the occurrence of irAEs, time to autoantibody appearance and number of positive autoantibodies included in the panel at week 48 after the initiation of ICI therapy.

### Study Design and Ethical Considerations

The protocol of the AUTENTIC study was designed in accordance with the Strengthening the Reporting of Observational Studies in Epidemiology guidelines. Accordingly, a multicenter observational prospective cohort study was designed with the participation of the following hospitals of the north of Spain: Araba University Hospital (Vitoria-Gasteiz), Navarra University Hospital (Pamplona), Galdakao University Hospital (Galdácano), Donostia University Hospital (San Sebastián) and San Pedro University Hospital (Logroño).

This study will be conducted in accordance with the Good Clinical Practice guidelines of the International Council for Harmonization (ICH) E6, the principles of the Declaration of Helsinki, and local regulations. Informed consent will be obtained from all participants before their enrollment. The study protocol has been reviewed and approved by the Ethical Committee of the Basque Country [code: PI2018106 (EPA-SP)] and the Spanish Agency of Medicines and Medical Devices (code: ILB-NIV-2018–01). The study has been registered in ClinicalTrials.gov (NCT03868046).

### Setting and Duration

The current study will consecutively enroll patients diagnosed with different types and stages of solid organ malignant tumors who are candidates for treatment with ICIs. In the setting of outpatient clinics, patients will be recruited by the medical oncologists responsible for their care in one of the participating hospitals. Recruitment will continue until the desired sample size is reached (see “*Study size calculation*” section). The autoantibody panel under study will be performed when the whole cohort reaches week 48 of follow-up from the time of inclusion, which will coincide with the ICI initiation date. Patients lost to follow-up will be censored at the time of their last follow-up.

### Participants

All patients diagnosed with a solid organ malignant tumour of any stage amenable to ICI therapy according to current guidelines are potentially eligible to participate. Patients will be included by the medical oncologists in charge in the outpatient clinic setting.

The patients must meet all the following inclusion criteria:

1) Initiation of treatment with an ICI or a combination of ICIs
2) ICI naïve status, though those treated in the past with other systemic therapies for cancer, such as chemotherapy or tyrosine-kinase inhibitors, may be included
3) Provision of written informed consent.

Exclusion criteria will be the following:

1) Life expectancy of less than 3 months from the start of ICI therapy
2) Contraindication to ICI therapy: proven hypersensitivity or history of anaphylactic allergic reactions secondary to ICIs, active severe autoimmune disease, or poor performance status (Eastern Cooperative Oncology Group score ≥ 3)
3) Active immunosuppressive treatment: prednisone at doses >10 mg/day or equivalent (e.g., 1.5 mg/day of dexamethasone), azathioprine, methotrexate, mycophenolate, cyclophosphamide, rituximab, leflunomide, anti-tumor necrosis factor agents (infliximab, etanercept, adalimumab, golimumab), or abatacept, among others.

### Follow-Up Methods

Once enrolled in the study, patients will be monitored following standard recommendations. In accordance with clinical practice guidelines (Schneider et al., 2021; Schneider et al., 2022), all patients will be assessed in a treatment planning visit at baseline. In this initial visit, attending physicians will take a detailed personal and family history focused on ADs, chronic infectious and organ-specific diseases, and conduct a physical examination. In addition, to guide their care, patients will undergo a baseline blood test including a complete blood count, measurement of glucose and thyroid stimulating hormone (TSH) levels and lipid profile, renal and liver function tests, coagulation tests, and serological tests for hepatitis B and C viruses and HIV.

The frequency of follow-up will depend on the treatment schedule of each specific ICI, discretion of the attending physician and occurrence of complications (irAEs, non-immune-related toxicity, infections). All patients will have direct telephone access to the investigators in order to report early symptoms potentially related to the occurrence of irAEs*.* Treatment schedules of the ICIs will be classified into every 2 or 3 weeks up to week 12; thereafter, drug administration may be spaced out to every 4 or every 6 weeks, respectively, according to the drug data sheets.

**FIGURE 1:**
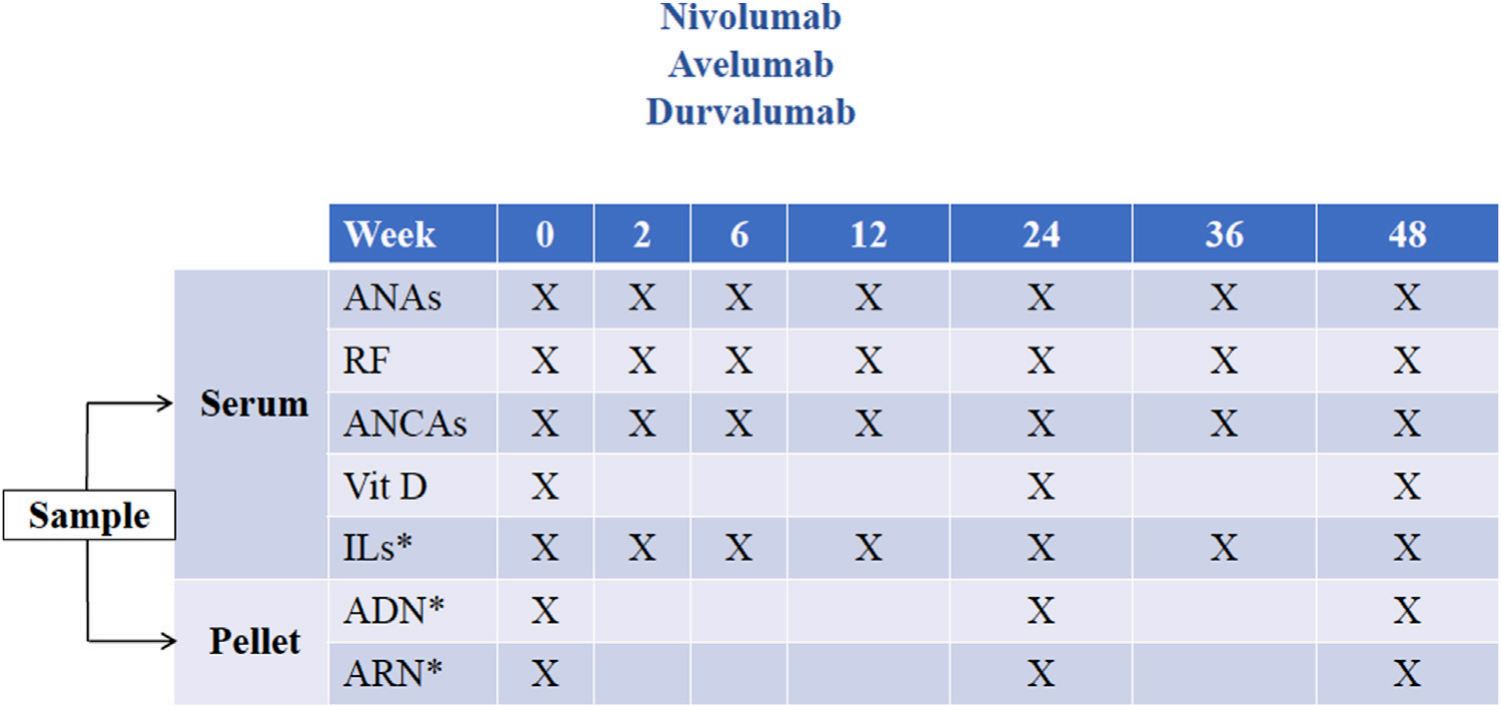
Study schedule 1. Timing of ordinary samples for patients included receiving immune checkpoint inhibitors administered every 2 weeks (nivolumab, avelumab, and durvalumab). *Measurements not included in this study (potentially for use in future research projects). ANA, antinuclear antibody; RF, rheumatoid factor; ANCA, antineutrophil cytoplasmic antibody; Vit D, vitamin D (measured as 25-hydroxycholecalciferol); IL, interleukin.

**FIGURE 2:**
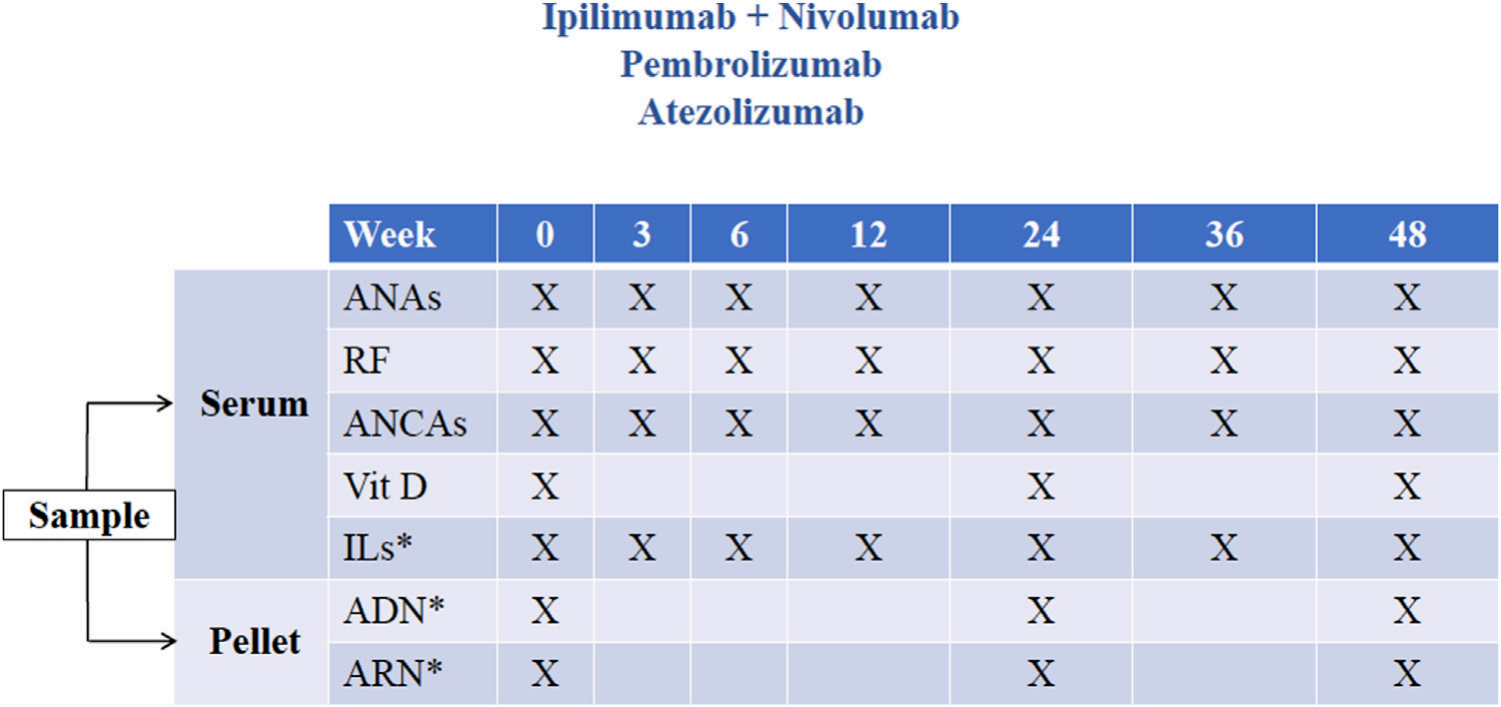
Study schedule 2. Timing of ordinary samples for patients included receiving immune checkpoint inhibitors or combinations of immune checkpoint inhibitors administered every 3 weeks (ipilimumab + nivolumab, pembrolizumab and atezolizumab). *Measurements not included in this study (potentially for use in future research projects). ANA, antinuclear antibody; RF, rheumatoid factor; ANCA, antineutrophil cytoplasmic antibody; Vit D, vitamin D (measured as 25-hydroxycholecalciferol); IL, interleukin.

Two types of blood samples will be taken for research purposes only: ordinary samples, defined as those drawn routinely as scheduled (Figure 1 and Figure 2), and extraordinary samples, defined as those taken at the time an irAE is detected. As far as possible, ordinary blood samples will be collected at the same time as blood draws for medical purposes before the administration of each ICI dose, in accordance with the study schedules (Figures 1, 2).

Ordinary and extraordinary samples will be analyzed together at the end of the follow-up period, once the whole cohort reaches week 48.

#### Ordinary Samples and Study Schedules

Prior to receiving the first dose of ICI, all patients will undergo a baseline (i.e., week 0) blood test for research purposes, at the same time as their baseline blood test for medical purposes.

For ICIs administered every 2 weeks (nivolumab, avelumab, and durvalumab), sequential blood tests will be performed per protocol at weeks 2, 6, 12, 24, 36 and 48 after ICI initiation, all of them at the same time as draws for medical purposes (Figure 1). In other words, four ordinary samples will be obtained in the first 12 weeks (i.e., at 0, 2, 6, and 12 weeks after ICI initiation). Thereafter, the ordinary blood sampling interval will be every 12 weeks up to the end of follow-up at week 48.

For ICIs or combinations of ICIs administered every 3 weeks (ipilimumab plus nivolumab, pembrolizumab, and atezolizumab), sequential blood tests will be performed at weeks 0, 3, 6, 12, 24, 36 and 48 after ICI initiation, all of them at the same time as draws for medical purposes (Figure 2). In other words, four ordinary samples will be obtained in the first 12 weeks (i.e., at 0, 3, 6, 12 weeks after ICI initiation). Thereafter, the ordinary blood sampling interval will be 12 weeks up to the end of follow-up at week 48.

In the event of a delay in the administration of an ICI dose due to any eventuality other than an irAE, the ordinary sample corresponding to that treatment cycle will not be obtained. If the delay is due to the occurrence of an irAE, then an extraordinary sample will be obtained, as per protocol (see “*Extraordinary samples*” in the next section). Once treatment resumes, the ordinary sample corresponding to that treatment cycle will be collected per protocol.

#### Extraordinary samples

At the time an irAE is detected, an additional blood sample will be collected for tests including the autoantibody panel under study. At the discretion of the attending physician, screening for other more specific antibodies may be requested based on the clinical manifestations of the patient. For ethical and decision-making reasons, the results of these extraordinary analyses for medical purposes will be available to the attending physicians.

### Laboratory Management and Sample Circuit

Both ordinary and extraordinary samples for research purposes will be separated from samples for medical purposes and will be processed to obtain six 500-μL aliquots of serum each. These aliquots will be stored at −80°C and appropriately labelled and coded in the biobank of each participating center. Ordinary samples corresponding to baseline (week 0), week 24 and week 48 will be processed to obtain peripheral blood mononuclear cells (PBMCs), which will be stored in the biobank at −80°C as cell pellets for subsequent DNA and RNA extraction.

After completion of follow-up, serum samples will be centralized in a single autoimmunity laboratory located in the Central Laboratory of Araba University Hospital (Vitoria-Gasteiz) to be analyzed by a blinded team of physicians and technicians with extensive experience in clinical laboratory testing. The panel of autoantibodies under study (ANAs, RF, and ANCAs) will be used to screen the serum from the ordinary and extraordinary samples. In some cases, the serum from the extraordinary samples obtained at the time of an irAE diagnosis may be tested for other more specific autoantibodies, at the discretion of the attending physician. In addition, vitamin D levels will be measured as 25-hydroxycholecalciferol in the serum from ordinary samples at baseline and weeks 24 and 48.

### Patient Safety

The E6(R1) Guidelines for Good Clinical Practice of the International Council for Harmonization of Technical Requirements for Pharmaceuticals for Human Use defines an adverse event as any unfavorable and unintended sign (including an abnormal laboratory finding), symptom or disease, whether or not related to the investigational product. This includes events related to the product, or comparator, or to the procedures involved.

If a patient develops an irAE, the attending physician will proceed according to clinical practice guidelines (Brahmer et al., 2021). Depending on the severity of the irAE, the maintenance and temporary or permanent ICI withdrawal will be considered. Toxicity will be assessed individually for each specific irAE type.

### Variables

Patients included in the study will be identified by a numerical code. In this way, the data collected by researchers will not include personal information, safeguarding patient confidentiality. The transmission, processing, and dissemination of personal data from all the participating patients will be handled in accordance with Regulation (EU) 2016/679 of the European Parliament and of the Council of 27 April 2016 on the protection of natural persons regarding the processing of personal data and the free movement of such data, i.e., the General Data Protection Regulation.

Study data will be entered into an electronic database to which all participating hospitals will have centralized access, and which will comply with all data protection requirements.

Dependent variable: occurrence of irAEs.

An irAE is defined as any symptom, sign, syndrome, or disease caused by an immune-activating mechanism during the administration of an ICI once other causes such as an infectious disease or tumor progression have been ruled out.

Other descriptive variables related to irAEs:

• Description of symptoms related to the irAE
• Type: organ-specific or systemic (≥ 2 organs affected)
• Description of the involved organ
• Syndrome/disease: Sjögren’s syndrome, sarcoidosis, systemic vasculitis, systemic lupus erythematosus, etc.
• Severity, according to Common Terminology Criteria for Adverse Events v. 5.0
• Date of diagnosis of the irAE
• Treatment used for the irAE: non-steroidal anti-inflammatory drugs, glucocorticoids, immunosuppressive drugs other than glucocorticoids
• ICI dose reduction: yes/no
• ICI withdrawal: yes/no; if yes: temporary/permanent.

Independent variables: the following potential predictors of irAEs will be collected and analyzed: Epidemiological data: date of birth, sex, ethnicity.

General clinical data:

• Smoking and alcohol use
• Comorbidities: arterial hypertension, hypercholesterolemia, diabetes mellitus, cirrhosis/liver failure, renal insufficiency, thyroid dysfunction
• Chronic infections: hepatitis B, hepatitis C, HIV infection
• Autoimmune diseases prior to ICI initiation
• Type of previous autoimmune disease (description)
• Eastern Cooperative Oncology Group performance status score
• Body mass index (in kg/m^2^).

Data related to the tumour:

• Tumour type
• Date of diagnosis
• PD-L1 expression in tumour cells (%)
• Date of initiation of ICI therapy
• Dates of ICI administration
• Date of progression
• Date of death
• Progression-free survival, defined as time from start of ICI therapy to tumor progression, death or last day of follow-up
• Overall survival, defined as time from start of ICI therapy to death or the last day of follow-up
• Cause of death: cancer-related, non-cancer-related, ICI-associated toxicity (immune-related, non-immune-related adverse event, infection or other).

Data related to treatment received before ICI initiation:

• Nonsteroidal anti-inflammatory drugs
• Glucocorticoids
• Other immunomodulatory drugs: e.g., colchicine, hydroxychloroquine, mesalazine, or sulfasalazine
• Oral anticoagulants (acenocoumarol, low-molecular-weight heparin, direct oral anticoagulants).

Data related to ICI:

• Type: anti-CTLA4 (ipilimumab), anti-PD1 (nivolumab and pembrolizumab), anti-PD-L1 (atezolizumab, avelumab, and durvalumab)
• Monotherapy or combination therapy (description)
• Adjuvant or palliative therapy, and if palliative, line of therapy (first line, second line, other).

Laboratory data:

Obtained from blood samples for care and research purposes:

• Glomerular filtration rate (ml/min/m^2^), levels of glucose (mg/dl), bilirubin (mg/dl), alanine aminotransferase, aspartate aminotransferase, gamma-glutamyltransferase (U/L), and thyroid stimulating hormone (μUI/mL).
• Prothrombin time (percentage) or international normalized ratio (INR)
• Complete cell count: haemoglobin (g/dl), white blood cells (/µL), differential white blood cell count of neutrophils, lymphocytes, monocytes, and eosinophils (/μL) and platelets (/μL)
• Lipid profile: levels of cholesterol, triglycerides, and high- and low-density lipoprotein cholesterol (mg/dl).

Obtained from blood samples only for research purposes:

• 25-hydroxycholecalciferol (ng/ml) at weeks 0, 24 and 48.
• Levels of the autoantibodies under study (i.e., ANAs, RF and ANCAs) in ordinary samples according to study schedules (Figures 1, 2) and extraordinary samples.

### Management of Confounding and Interaction Factors

The use of glucocorticoids for an indication other than treatment of an irAE will be considered a confounding factor. To control for this source of confounding, the dose and duration of glucocorticoid therapy will be minimised, and multivariate analysis will be adjusted for concomitant glucocorticoid use.

The coexistence of an autoimmune disease with cancer will be considered a potential effect modifier (i.e., interaction). Furthermore, female sex is known to be a risk factor for ANA positivity and high ANA titers (Li et al., 2011). To facilitate the interpretation of these two potential interactions, data will be presented for patient subgroups in the form of contingency tables.

### Data Sources and Measurements

As mentioned above, for ANA, RF and ANCA measurements, all the samples will be centralized in the autoimmunity laboratory of the Central Laboratory of the Araba University Hospital (Vitoria-Gasteiz). The measurements will be carried out by two blinded technicians with accredited experience in the field of autoimmunity tests.

In accordance with current recommendations (Agmon-Levin et al., 2014), ANA detection will be performed using IFAs on a fully automated system (EUROPattern, EUROIMMUN), using a combination of HEp-2 cells and rodent tissue sections as a dual substrate. If IFA results are positive in one or more screening dilutions, the serum will be serially diluted and re-analyzed until fewer than half of the cells on the slide produce detectable fluorescence; the ANA titer will be reported as the dilution prior to the point at which this occurs. Antinuclear antibodies will be considered positive from a titer ≥1:40 on rodent tissue and ≥1:80 on HEp-2 cells. Positivity will be classified as low or high based on ANA titers between 1:40 and 1:160, and ≥1:320, respectively.

Quantitative measurement of RF will be performed on serum samples with the ARCHITECT automated analyzer (Abbott) (Dupuy et al., 2014), based on a latex agglutination immunoturbidimetry technique using an antigen-antibody reaction between RF present in the sample and denatured human immunoglobulin isotype G adsorbed on latex particles. The resulting agglutination is detected as a change in absorbance (572 nm), and the magnitude of the change is proportional to the amount of RF in the sample. The concentration is estimated by interpolation on a calibration curve prepared from calibrators of known concentration. The result will be considered positive if the RF value is greater than 30 IU/ml.

Testing of ANCAs will be performed using an IFA in a fully automated system (EUROPattern), and results will be considered positive for titers ≥ 1:20. Borderline and positive samples will be retested with myeloperoxidase and proteinase-3 immunoassays from Thermofisher® on the UNICAP 250 analyser using fluoroimmunoassay technology.

### Management of Potential Bias

The inclusion of patients with previous ADs could be influenced by attending physicians’ concern about inducing irAEs, and this could lead to a biased representation of the population eligible for the study. Researchers will encourage oncologists to include patients with ADs in remission on a case-by-case basis.

To minimize a detection bias arising from a higher level of suspicion of irAEs in autoantibody-positive patients, the autoantibody panel under study will be performed and the results analyzed after the whole cohort reaches week 48 of follow-up. On the other hand, for ethical and practical reasons, the autoantibody results from the extraordinary samples taken for medical purposes will be available to the attending physicians. Nonetheless, the laboratory staff involved in the detection and interpretation of autoantibodies will always be unaware of the clinical status of each patient.

### Sample Size Calculation and Statistical Analysis

To estimate an expected sensitivity of the autoantibody panel under study of 0.90 with a 95% confidence interval of not less than 0.75, 70 cases with irAEs will be needed. Considering irAEs associated with ICIs will develop in 25% of cases (Wang et al., 2019), a total of 210 patients without irAEs will be required. Considering a loss to follow-up of 5%, we should recruit a total of 294 patients.

Regarding the statistical analysis, firstly, a description of the cohort will be performed. Quantitative variables will be reported as mean ± standard deviation or median (interquartile range) and compared between groups using Student’s t-test for unpaired data or the Mann-Whitney U-test, depending on whether data follow a normal distribution. Categorical variables will be expressed as frequencies (percentages) and compared between groups using Pearson’s Χ^2^ or Fisher’s exact test based on the expected frequency of cells. To assess the primary endpoint of the study (i.e., proportion of patients developing at least an irAE), the autoantibody panel will be considered positive when any of the autoantibodies included in the panel is positive according to the following cut-off points: ANA titers ≥ 1:40, ANCA titers ≥ 1:20 or RF value ≥ 30 IU/ml. The autoantibody panel and the titers of autoantibodies will be handled as a categorical variable (namely, positive or negative result) and as a quantitative variable, respectively. It is planned to perform a subgroup analysis depending on whether or not patients have an AD before their inclusion. An analysis of variance for repeated measures will be performed to explore the profile of detectable autoantibodies over the 48 weeks of follow-up.

Survival will be assessed using a Kaplan-Meier analysis and compared between groups using the log-rank Mantel-Cox test. A subgroup analysis according to tumour type and extent of cancer (local, advanced, and metastatic disease) will be performed to assess survival outcomes*.* A multivariate analysis using logistic regression and Cox regression will be performed to detect factors associated with the occurrence of irAEs. Applying a manual backward stepwise procedure, a multivariate model will be constructed from variables that either have a p-value of less than 0.10 in the univariate analysis or are considered relevant to the dependent variable on the basis of previous studies (e.g., sex and age of patients, type of ICI). Variables not reaching statistical significance will be dropped from the final predictive model. The sensitivity, specificity, positive predictive value, negative predictive value, and probability ratios with their corresponding 95% confidence intervals of the model will be calculated. The diagnostic accuracy of the predictive model inferred from the logistic regression model will be described with the area under the receiver operating characteristic curve.

Two-sided hypothesis tests will be performed, and the significance level will be set at 5%. The statistical analysis will be conducted using IBM SPSS Statistics software version 27.0 (Chicago, IL).

## Discussion

The progressive adoption of immunotherapy in various types of cancer and the growing trend towards the use of combinations of ICIs are extending the problem of irAEs to a larger number of patients (Ramos-Casals et al., 2020). A better understanding of the pathophysiology of irAEs would allow us to select more efficient clinical, analytical, and histological markers for the early and accurate detection of immune-mediated toxicity (Patil et al., 2018). To date, several immunological mechanisms have been proposed to explain the development of irAEs in cancer patients treated with ICIs: direct molecular mimicry, abnormal cytokine production, the influence of environmental factors such as the gut microbiome, activation of cytotoxic T cells, and activation of autoreactive B cells with secondary production of autoantibodies (Lee et al., 2021). The actual mechanism by which ICIs lead to autoantibody release has yet to be established. ICI-enhanced T-cell damage to tumour tissue could overexpress antigens that trigger B-cell activation (Thibult et al., 2013; Das et al., 2018). Alternatively, ICI-induced cytokines could indirectly activate B cells or their expansion into plasmablasts (Vazquez et al., 2015). In recent years, the potential use of autoantibody batteries as predictors of irAEs has become a promising and widespread field of research (Salim et al., 2021; Seki et al., 2021).

Nonetheless, there is limited evidence on the diagnostic performance of autoantibodies to predict the occurrence of irAEs. Given this, clinical practice guidelines do not currently recommend the measurement of autoantibodies before the initiation of ICI therapy (Kostine et al., 2021). Indiscriminate use of autoantibodies presumed to be diagnostic in patients treated with ICIs would cause an increase in false positive results and findings of doubtful clinical value, which could lead to confusion among treating physicians. Nevertheless, progress has been made in understanding the impact of pre-existing or ICI-induced autoantibodies on the risk of irAEs. This relationship between autoantibodies and toxicity is well described in the case of organ-specific irAEs and the autoantibodies typically associated with such events (Mammen et al., 2019; Hasan Ali et al., 2020), especially when there is a high rate of seropositivity. For instance, the risk of developing nivolumab-destructive thyroiditis is known to be significantly higher in patients with pre-treatment anti-thyroid antibodies (namely, anti-peroxidase and anti-thyroglobulin antibodies) (Osorio et al., 2017; Kobayashi et al., 2018; Basak et al., 2020). However, the heterogeneity and complexity of irAEs, which may resemble any described AD, together with the variable seropositivity of the corresponding autoantibodies, make it difficult to develop autoantibody panels sensitive enough for screening purposes.

This difficulty in implementing diagnostically efficient autoantibody panels in clinical practice raises the question of whether screening for ADs is justified. In the general asymptomatic population, the indiscriminate use of autoantibody testing for early diagnosis of ADs is not indicated due to the high rate of false positive results; but could screening for irAE risk be warranted in patients who are going to receive ICIs? In fact, in some complex ADs, such as systemic lupus erythematosus, there is an early appearance and aggregation of autoantibodies, starting with generic autoantibodies and progressing to more specific ones, several years before disease onset (Arbuckle et al., 2003). This anticipatory phenomenon has also been observed in other more organ-specific ADs, such as primary biliary cirrhosis, in which anti-mitochondrial antibodies appear in advance of clinical manifestations (Jones, 2000). Since immunotherapy has been likened to releasing the brake on the immune system, it could be hypothesized that ICIs may accelerate this process of early emergence of autoantibodies.

If we were to accept that autoantibodies do have a role to play in the diagnosis of irAEs, the next question would be to elucidate the most efficient screening panel, which should perform with an optimal balance between sensitivity (i.e., high seropositivity rate) and specificity (i.e., low false positivity rate). This approach has led some authors to propose the targeted use of autoantibody testing depending on the specific type of irAE experienced by a patient (Ghosh et al., 2021), as in the thyroiditis model. Unfortunately, not all reported irAEs have been paired with a specific autoantibody. A priori, organ-specific autoantibodies are not useful for overall screening for irAEs, which relies on sensitive rather than specific diagnostic methods. Nonetheless, it has been shown that positivity for certain specific antibodies, such as ANAs, RF, and anti-thyroid antibodies, could be useful in screening for any type of irAE, regardless of the organ involved (Toi et al., 2019; Music et al., 2020). At this point, we could opt to use either very large (even massive) panels or “generic” autoantibody panels that can be applied cross-sectionally to most patients. The first option has the disadvantages of high cost, low availability, and complex interpretation: the greater the number of measurements, the greater the number of overlaps and false-positive or erratic results. With the launch of the AUTENTIC project, our group is seeking to advance the second option, with the development of a generic and routinely accessible panel of autoantibodies consisting of ANAs, RF, and ANCAs.

To date, research evaluating the diagnostic value of generic autoantibodies has differed in design, the battery selected, the target population and the main results (Table 1). Toi et al. conducted a retrospective study of 137 patients diagnosed with non-small cell lung carcinoma treated with nivolumab or pembrolizumab who were tested for ANAs, RF and anti-thyroid antibodies at baseline (Toi et al., 2019). Patients seropositive for any autoantibody, but especially for RF and ANAs, were found to be at increased risk of developing irAEs. These results were not confirmed, however, by two retrospective studies with smaller sample sizes by Yoneshima et al. and Sakakida et al., which also assessed ANAs as markers of the risk of immune-related toxicity (Yoneshima et al., 2019; Sakakida et al., 2020).

**TABLE 1:** Summary of the studies assessing generic autoantibodies as predictors of immune-related adverse events in patients on immune checkpoint inhibitors.

Study and reference	Design	Sample size	Type of immune checkpoint inhibitor	Pan-tumor	Autoantibody panel (status assessed^a^)	Main results
Toi et al. (2019) ^108^	Retrospective Single center	137	Nivolumab or Pembrolizumab	No (NSCLC)	ANA, RF, and ATA (preexisting)	Autoantibodies were associated with: a higher risk of irAEs (OR 3.25, *p* = 0.001) and a longer PFS (HR 0.53, *p* = 0.002)
Yoneshima et al. (2019) ^110^	Retrospective Single center	83	Nivolumab or Pembrolizumab	No (NSCLC)	ANA (preexisting)	ANAs were not associated with irAEs, though the risk of irAEs tended to be higher with higher titers of ANAs. ANAs were associated with: a shorter PFS (HR 2.06, *p* = 0.02) and a shorter OS (HR 2.31, *p* = 0.03)
Sakakida et al. (2020) ^111^	Retrospective Single center	191	Nivolumab, Pembrolizumab, Atezolizumab or Durvalumab	Yes	ANA (preexisting)	ANAs were not associated with irAEs, except for colitis (22 vs. 1.6%, *p* = 0.002). ANAs were not associated with ORR or DCR.
De Moel et al. (2019) ^112^	Retrospective Two centers	133	Ipilimumab (100% of patients), Pembrolizumab or Nivolumab (49.6% of patients)	No (melanoma)	ANA, anti-dsDNA antibody, ENA^b^, RF, ACPA, ASMA, AMA, anti-LKM antibody, and ATA (development)	Autoantibodies were associated with: a trend for higher risk of irAEs (OR 2.92, *p* = 0.12) and for better OS (HR 0.66, *p* = 0.21) ATAs were associated with: higher ORR (OR 5.43, *p* = 0.021)
Giannicola et al. (2019) ^113^	Retrospective Multicenter	92	Nivolumab	No (NSCLC)	ANA, ENA^c^,and ASMA (short-term development, within 30 days)	Early detection of autoantibodies was associated with: a higher risk of irAEs (HR not available, *p* = 0.002), a higher PFS (HR 0.23, *p* = 0.004) and a higher OS (HR 0.28, *p* = 0.030)
Les et al. (2021) ^114^	Retrospective Single center Pilot-study	69	Nivolumab	Yes	ANA, RF, and ANCA (preexisting and development)	Autoantibodies were associated with a higher risk of irAE (OR 46.61, *p* = 0.010)
AUTENTIC	Prospective Multicenter	294	All approved immune checkpoint inhibitors	Yes	ANA, RF, and ANCA (preexisting and development)	-

a Status assessed: preexisting antibodies (at baseline), and/or the development of antibodies (during treatment).

b Using U1RNP, SS-A/Ro, SS-B/La, centromere B, Scl-70, Jo-1, and Sm proteins as antigens.

c The antigens used in this panel were not specificed by the study authors.

Taking a different approach, de Moel et al. designed a retrospective study that included 133 patients with melanoma, all of them treated with ipilimumab, and from whom pre- and post-treatment samples were obtained (De Moel et al., 2019). In this case, the autoantibody battery selected was broader than in previous studies, consisting of 23 different autoantibodies including anti-DNA, anti-smooth muscle, anti-mitochondrial, anti-liver-kidney microsomal, anti-peroxidase, and anti-thyroglobulin antibodies, as well as ANAs, RF, ACPAs, and ENAs (using U1RNP, SS-A/Ro, SS-B/La, centromere B, Scl-70, Jo-1, and Sm proteins as antigens). Among the 99 patients who were seronegative at baseline, a seroconversion frequency of as high as 19.2% was observed for some types of autoantibodies. The presence of autoantibodies before ICI initiation was not associated with the occurrence of irAEs. Nonetheless, a statistically non-significant association was observed between becoming positive for any autoantibody and the occurrence of irAEs during follow-up (OR = 2.92; 95% confidence interval = 0.85–10.01) and the association became significant when the analysis focused on specific irAEs [arthralgias/arthritis, hepatitis, thyroid dysfunction, colitis, adrenal insufficiency, dermatitis, or dry syndrome (Sjögren’s syndrome)] related to the battery under study. Interestingly, no association was observed between each type of organ-specific irAE and its corresponding specific autoantibody. The value of autoantibody seroconversion was also highlighted by Giannicola et al., who found that ANA, ENA, or anti-smooth muscle antibody positivity after nivolumab initiation was associated with a higher risk of irAEs in patients with non-small cell lung cancer (Giannicola et al., 2019).

Within the AUTENTIC project, we have recently published a retrospective, single-center, pilot study showing a potential association between autoantibody positivity, considering an autoantibody battery consisting of ANAs, RF, ANCAs and anti-thyroid antibodies, and the occurrence of irAEs in 69 patients treated with nivolumab (Les et al., 2021). A hallmark of that study is that we included patients diagnosed with various types of cancer, non-small cell lung cancer being the most common. Overall, 37.5% of the cohort experienced at least one irAE. We observed that the autoantibody panel positivity was significantly higher in patients experiencing irAEs (87.5 vs. 30% in those not experiencing irAEs, *p* = 0.009). Consistent with previous evidence, other factors that could influence the appearance of irAEs were young age and female sex. The statistical model based on sex, age at ICI initiation and positivity for any autoantibody included in the battery predicted the occurrence of irAEs with an area under the receiver operating characteristic curve of 90.6%. In the logistic regression model, the only independent predictive factor of irAEs was positivity in the autoantibody panel under study (OR = 46.61, *p* = 0.010). Furthermore, a marked overlap was demonstrated between the different autoantibodies under study.

Besides these studies assessing some routinely available autoantibody panels, other approaches that apply throughput autoantigen array technology should be considered. For example, using serological analysis of recombinant cDNA expression, a technique designed to detect tumor antigens, Tahir et al. found anti-GNAL and anti-ITM2B autoantibodies correlated with hypophysitis and anti-CD74 antibodies correlated with pneumonitis (Tahir et al., 2019). Further, applying a human proteome microarray system called HuProt, Gowen et al. identified a specific pre-treatment autoantibody profile in the serum of patients, which was associated with severe irAEs (Gowen et al., 2018).

Despite significant advances, currently available research on the role of autoantibodies in the irAE diagnosis has several limitations. Firstly, there is a lack of prospective studies assessing autoantibody status over time and the timing of autoantibody emergence. Further long-term studies are needed, as there is already evidence that the dynamic of irAE onset differs depending on the ICI administered and the organ involved. Another shortcoming is that most publications focus on a specific type of cancer, and hence, a pan-tumour perspective is lacking. Indeed, the first tumour-agnostic approval of pembrolizumab for the treatment of patients with deficient DNA mismatch repair and high microsatellite instability, regardless of the origin of the cancer, makes the analysis of pan-tumour cohorts even more interesting and necessary (Marabelle et al., 2020). In addition, few studies have assessed the impact of different types of ICIs, the combination of anti-CTLA4 and anti-PD1/PDL1 drugs, and the combination of ICIs with chemotherapy or other targeted antitumour drugs on irAEs. Recently, the benefit of combining nivolumab and relatlimab, a new ICI that blocks the lymphocyte-activation gene (LAG-3), has been shown in patients with advanced melanoma (Tawbi et al., 2022). In the coming years, it is expected that the combinations of different families of ICIs will increase in number and complexity, encompassing multiple molecular targets and therapeutic indications.

In the face of this increasing complexity, one reasonable approach would be to establish prospective cohorts and biobanks, allowing multi-center collaboration and the storage of specimens that can answer our questions in the future. At the patient level, the optimal management of irAEs should promote cross-specialty networking. In this context, we believe that the AUTENTIC project is a useful step in tackling the challenge of irAEs in patients on ICIs, with the leading role it gives to biobanks and its wide scope (broad inclusion and few exclusion criteria) spanning adverse effects that affect several organ systems and require the involvement of numerous medical specialties.

## Abbreviations

ACPA, anti-citrullinated peptide antibody; AMA, anti-mitochondrial antibody; ANA, antinuclear antibody; ANCA, antineutrophil cytoplasmic antibody; anti-LKM, anti-liver-kidney microsomal; ASMA, anti-smooth muscle antibody; ATA, antithyroid antibody; DCR, disease control rate; dsDNA, double-stranded DNA; ENA, extractable nuclear antigen antibody; HR, hazard ratio; irAE, immune-related adverse event; NSCLC, non-small cell lung cancer; OS, overall survival; OR, odds ratio; PFS, progression free survival; ORR, overall response rate; RF, rheumatoid factor.
